# Comparative analysis of genome sequences from four strains of the *Buchnera aphidicola* Mp endosymbion of the green peach aphid, *Myzus persicae*

**DOI:** 10.1186/1471-2164-14-917

**Published:** 2013-12-24

**Authors:** Zhijie Jiang, Derek H Jones, Sawsan Khuri, Nicholas F Tsinoremas, Tania Wyss, Georg Jander, Alex C C Wilson

**Affiliations:** 1 Center for Computational Science, Miller School of Medicine, University of Miami, Coral Gables 33146, FL, USA; 2 Department of Biology, University of Miami, Coral Gables 33146, FL, USA; 3 Department of Computer Science, University of Miami, Coral Gables 33146, FL, USA; 4 Department of Medicine, Miller School of Medicine, University of Miami, Miami 33136, FL, USA; 5 Boyce Thompson Institute for Plant Research, Ithaca 14853, NY, USA

**Keywords:** *Buchnera aphidicola*, *Myzus persicae*, Aphid, Genome, Symbiont, Queuosine, Asparaginase

## Abstract

**Background:**

*Myzus persicae*, the green peach aphid, is a polyphagous herbivore that feeds from hundreds of species of mostly dicot crop plants. Like other phloem-feeding aphids, *M. persicae* rely on the endosymbiotic bacterium, *Buchnera aphidicola* (*Buchnera* Mp), for biosynthesis of essential amino acids and other nutrients that are not sufficiently abundant in their phloem sap diet. Tobacco-specialized *M. persicae* are typically red and somewhat distinct from other lineages of this species. To determine whether the endosymbiotic bacteria of *M. persicae* could play a role in tobacco adaptation, we sequenced the *Buchnera* Mp genomes from two tobacco-adapted and two non-tobacco *M. persicae* lineages.

**Results:**

With a genome size of 643.5 kb and 579 predicted genes, *Buchnera* Mp is the largest *Buchnera* genome sequenced to date. No differences in gene content were found between the four sequenced *Buchnera* Mp strains. Compared to *Buchnera* APS from the well-studied pea aphid, *Acyrthosiphon pisum*, *Buchnera* Mp has 21 additional genes. These include genes encoding five enzymes required for biosynthesis of the modified nucleoside queosine, the heme pathway enzyme uroporphyrinogen III synthase, and asparaginase. Asparaginase, which is also encoded by the genome of the aphid host, may allow *Buchnera* Mp to synthesize essential amino acids from asparagine, a relatively abundant phloem amino acid.

**Conclusions:**

Together our results indicate that the obligate intracellular symbiont *Buchnera aphidicola* does not contribute to the adaptation of *Myzus persicae* to feeding on tobacco.

## Background

Intraspecific variation in host plant use is common and well-documented in aphids [e.g. [1-5], and recently reviewed by [6]. One factor that has been demonstrated to greatly impact host plant use in the pea aphid, *Acyrthosiphon pisum*, is the presence of the secondary bacterial symbiont, *Regiella insecticola*[7-9]. In an elegant study that first eliminated *R. insecticola* from a naturally infected *A. pisum* lineage and then re-introduced infection of *R. insecticola* to the cured lineage, Tsuchida and colleagues demonstrated that the ability of *A. pisum* genotype TUt to utilize white clover (*Trifolium repens*) as a host is greatly facilitated by *R. insecticola* infection yet see [10]. Whereas secondary bacterial symbionts are commonly associated with *A. pisum*, they are less commonly found in other aphid species, including the cosmopolitan and polyphagous green peach aphid, *Myzus persicae*[11]. However, like *A. pisum*, *M. persicae* possesses the obligate intracellular bacterial symbiont *Buchnera aphidicola*, which has been implicated in intraspecific variation of the host nutritional requirements [12].

Almost all aphids rely on *Buchnera* for the biosynthesis of essential amino acids that are not sufficiently abundant in phloem [13-15]. Whole genome sequencing of *Buchnera* from multiple aphid species demonstrated clear interspecific variation in the ability of *Buchnera* to synthesize amino acids [16-20]. For example *Buchnera* Sg from the monocot-feeding aphid *Schizaphis graminum* has accumulated mutations in five of the genes required for the reduction of sulfur and cysteine biosynthesis [17], thus constraining *S. graminum* to a diet with sufficient sources of fixed sulfur [21]. Consistent with this, interspecific variation in aphid performance on artificial diet provides strong evidence that, in a range of aphid species, host plant adapted lineages vary in their amino acid biosynthetic abilities [e.g. [12,22-24]. More recently, Vogel and Moran [12] identified six *A. pisum* lineages that varied in their dietary essential amino acid requirements. Using DNA sequencing and phenotyping of F1s from interclonal crosses they were able to demonstrate that, in the case of one of these six *A. pisum* lineages, dietary arginine requirements were determined by a mutation in the *argC* gene of its *Buchnera* endosymbiont.

*M. persicae* is a phloem-feeding insect that is found in all temperate regions of the world and infests hundreds of mostly dicot plant species, including many food and ornamental crops [25]. Although, as a species, *M. persicae* has a very broad host range, there are significant differences in host plant utilization and even specialization among *M. persicae* lineages [e.g. [1,26-29]. The best studied host plant specialized lineages of *M. persicae* are those that thrive on tobacco [30-32]. Typically, *M. persicae* lineages found on tobacco are red and have relatively high nicotine tolerance [33], whereas non-tobacco lineages are green through yellow in color and have lower tolerance to nicotine [34]. Although tobacco-adapted lineages have been shown to be morphologically and genetically distinct from non-tobacco lineages of *M. persicae*, other genetic evidence clearly demonstrates maintenance of gene flow between tobacco-adapted and non-tobacco lineages [27,35,36].

Motivated by the possibility that differences in host plant specialization in *M. persicae* correlate with differences in the metabolic capabilities of the primary endosymbiont we present here the whole genome sequence of *Buchnera aphidicola* Mp (*Buchnera* Mp), the previously unsequenced obligate intracellular symbiont of *M. persicae*, from two tobacco-adapted aphid lineages and two aphid lineages collected from pepper and potato, respectively. These three plant species belong to different genera in the Solanaceae family, and are therefore distinct, but nevertheless closely related. We find that, at 643,504 bp the *Buchnera* Mp genome is the largest of all fully-sequenced *Buchnera* genomes. In addition to the large circular chromosome, the *Buchnera* Mp genome includes the leucine (pLeu) and tryptophan (pTrp) biosynthesis plasmids that appear to be common to all *Buchnera* from the Aphidini [37]. Although the *Buchnera* Mp genome differs from other sequenced *Buchnera* genomes in the presence or absence of key genes, we found only limited within-species differences in DNA sequence and no differences in gene content between *Buchnera* Mp lineages in aphids collected from solanaceous tobacco and non-tobacco hosts.

## Results and discussion

### *Assembly of the four* Buchnera *Mp genomes and their associated plasmids*

We generated Roche 454 and Illumina sequence data for all four *Buchnera* Mp lineages. Total read number for the Roche 454 libraries, with an average read length of 218 bp, ranged from 72,240 – 86,041 (Table 1). Using a *de novo* assembly approach, more than 67% of all Roche 454 reads for each *Buchnera* Mp strain were assembled into contigs (Table 1). For each genome, the contigs homologous to *Buchnera* APS (PRJNA57805) were identified by BLAST search, and syntenic contigs were assembled into *Buchnera* Mp genome scaffolds that covered 90% of the anticipated genome of ~640 kbp – a size estimate derived from the genomes of *Buchnera* from *A. pisum* (PRJNA57805) and *Schizaphis graminum* (PRJNA57913). Gap regions in the genome scaffolds were filled using an overlap–consensus approach with the 36 bp single–end Illumina libraries that had generated 3,801,794 – 10,435,517 reads (Table 1 and see Materials and Methods). This overlap-consensus approach to gap filling was applied to 15 gaps in the *Buchnera* Mp USDA chromosome, 137 gaps in the W106 chromosome, 12 gaps in the F009 chromosome and 146 gaps in the G002 chromosome. Thus the draft genome sequences for each lineage comprised the joint assembly of Roche 454 reads and Illumina reads. Following assembly of closed bacterial chromosomes, closed leucine plasmids, and open tryptophan plasmids for each of the four *Buchnera* Mp lineages, we searched our assembled data for any contigs that might represent novel *Buchnera* plasmids or secondary bacterial symbionts. We did not find any evidence of novel plasmids or secondary symbionts in any of the assemblies.

**Table 1:** **Read and assembly statistics for each of the four *Buchnera *** **Mp genomes**

	** Tobacco**		** Non-tobacco**	
	**USDA**	**W106**	**F009**	**G002**
**454 reads**				
Total reads	72,648	86,041	72,240	83,404
Mapped reads	48,461	74,153	55,579	72,087
Mapped read rate	67%	86%	77%	86%
**Illumina reads**				
Total reads	8,191,608	5,030,809	3,801,794	10,435,517
Mapped reads	4,884,034	3,906,073	2,648,621	7,136,918
Mapped read rate	60%	78%	70%	68%
***Buchnera *** **genome assembly details**				
Number contigs >500 bp	48	152	36	184
Average size of contigs >500 bp	14,166	4,161	18,730	3,429
N50 of contigs >500 bp	62,925	6,320	123,327	5,580
Number of bases in contigs >500 bp	679,985	632,575	674,282	631,022
Total number contigs	1,556	3,420	1,810	3,709
N50	241	223	241	242
Number of bases	1,009,382	1,283,886	1,078,855	1,420,326
Size of largest contig	184,872	31,386	321,471	18,486
*Buchnera* syntenic contigs	15	137	12	146

The quality of the draft genomes was measured by coverage of Roche 454 reads and Illumina reads. The average of Roche 454 reads on the assembled genome sequences was 20×, while the average Illumina coverage was 200×. Surprisingly, some regions that were shared across all four *Buchnera* Mp strains were characterized by low read coverage. All of these low read coverage regions were poly-A rich regions. Roche 454 sequencing technology is unreliable for sequencing AT rich regions and thus, because of the poor quality of the 454 data it was difficult to map the Illumina data to these AT rich genomic regions. Thus, for genomic regions that had less than 21× Illumina coverage, we removed the 454 data, making a gap in the assembly that we then filled using the overlap-consensus applied above. Following this, we used Sanger sequencing to verify any low-coverage genomic regions. After validation, a final genome sequence was constructed for each *Buchnera* Mp chromosome and plasmid. These sequences have been deposited in Genbank under the following accessions: For the tobacco strains - BUMPUSDA,CP002697; BUMPUSDA_pLeu CP002698; BUMPUSDA_pTrp JF928419; BUMPW106 CP002699; BUMPW106_pLeu CP002700; BUMPW106_pTrp JF928420; for the strain from pepper - BUMPG002 CP002701; BUMPG002_pLeu CP002702; BUMPG002_pTrp JF928418; and for the strain from potato - BUMPF009 CP002703; BUMPF009_pLeu CP002704; BUMPF009_pTrp JF928417.

### *All four* Buchnera *Mp genomes contain an identical gene set*

**Figure 1:**
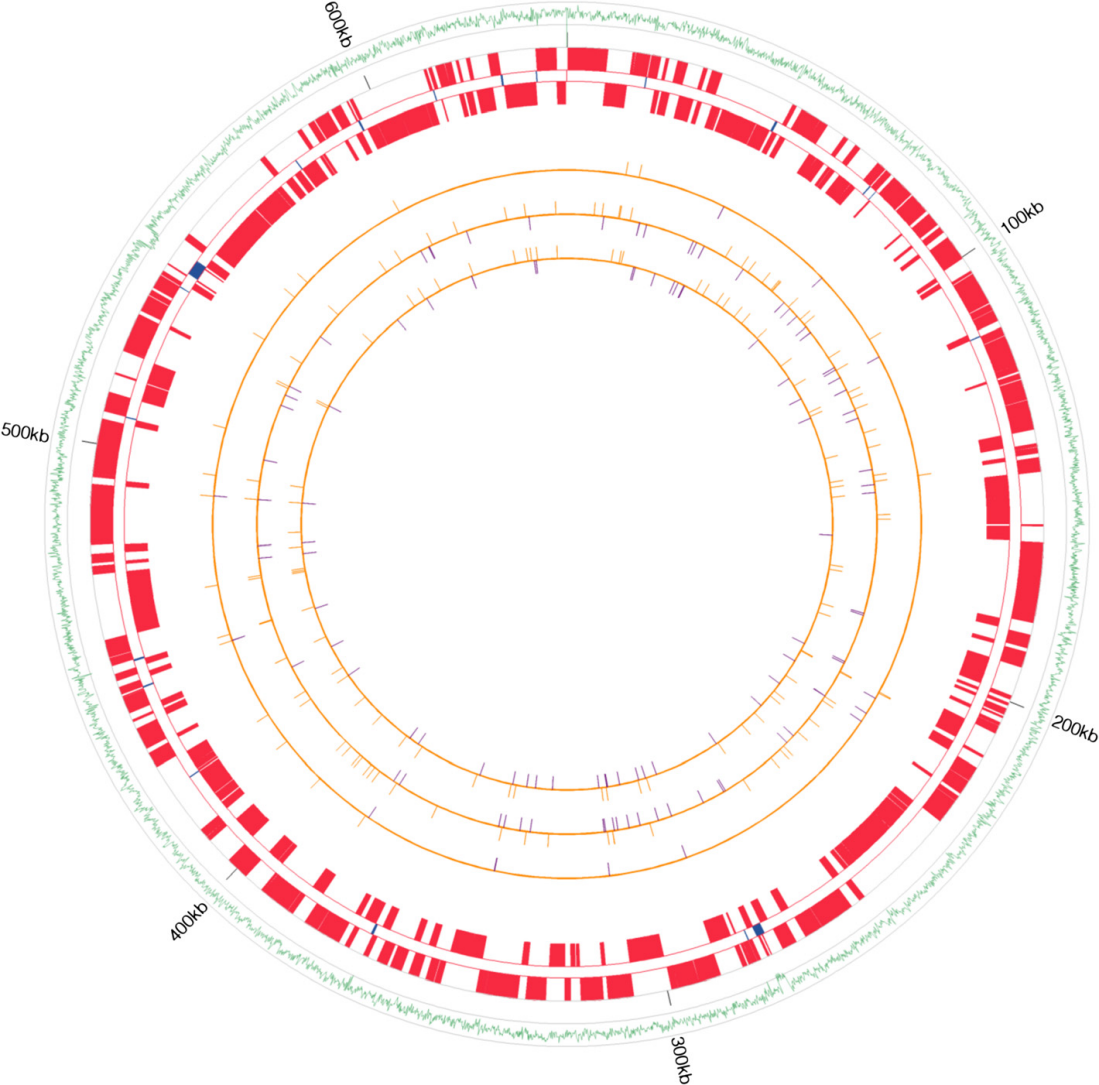
**Maps of *Buchnera *Mp USDA, W106, F009, and G002 strains.** The outside green wavy line is the AT frequency across the *Buchnera* Mp USDA genome. The three grey circles associated with the green wavy line mark 0, 0.5 and 1.0 AT frequency (from inside to outside). AT frequency was calculated using a 100 bp sliding-window with a window step of 10 bp. The red blocks on the two red circles are the annotated protein coding genes of the BuMp_USDA genome; the blue bars between the two red circles mark tRNAs and rRNAs. The three innermost orange circles represent the W106, F009 and G002 genomes with the orange bars on these circles indicating synonymous substitutions and the purple bars indicating nonsynonymous substitutions relative to BuMP_USDA.

This project was motivated by the possibility that differences in host plant specialization in *M. persicae* correlate with differences in the metabolic capabilities of the primary symbiont, *Buchnera* Mp. We found no differences in gene presence or absence across all four *Buchnera* Mp strains. Of the 582 protein coding genes in the tobacco adapted *Buchnera* Mp USDA genome, 312 are located on the positive strand, and 270 are on the negative strand. The relative positions of these genes are identical across all four *Buchnera* Mp strains. Differences in DNA sequence among the four strains are minimal (Table 2) with just under 13% of genes (74/581) containing nonsynonymous coding sequence differences in one or more of the four *Buchnera* Mp genomes (Figure 1, Table 3). We tested the distribution of nonsynonymous mutation on the genomes of *Buchnera* Mp strains W106, F009 and G002 by random statistical sampling [38] and cannot reject the hypothesis that nonsynonymous substitutions are randomly distributed (W106 p = 0.506, F009 p = 0.529 and G002 p = 0.522). Of the genes that contain nonsynonymous mutations, thirteen mutations affecting twelve genes are predicted by PROVEAN analysis [39] to have a deleterious effect on protein function (Additional file 1: Table S3). Of these thirteen genes, seven (*clpP*, *dnaE*, *mrcB*, *rpoB*, *ybgI*, *yggJ* and *uppS*) have functions that are internal to *Buchnera*, while the remaining five (*cmk*, *dut*, *fabD*, *pta* and *thrC*) are associated more directly with metabolic processes central to the symbiosis. Mutations in two genes are noteworthy. First, deleterious mutations in *ybgI* a cytoplasmic metal ion binding protein were identified in both non-tobacco adapted lineages (F009 and G002)(Additional file 1: Table S3 – marked by §). Second, the two non-tobacco adapted lineages share the same non-synonymous mutation in *thrC* (Additional file 1: Table S3 – marked by *). An amino acid biosynthesis gene, *thrC* is the final gene in the threonine biosynthesis pathway ([40] and see [41]). Threonine is one of the ten essential amino acids synthesized by *Buchnera*, suggesting that the non-tobacco lines of *M. persicae* examined here may have different threonine dietary requirements, an hypothesis that can be tested experimentally in future work.

**Table 2:** **Pairwise single nucleotide substitutions (SNPs) and insertions/deletions (Indels) among four *Buchnera *** **Mp strains**

	**USDA**	**F009**	**G002**	**W106**
USDA		185	183	51
F009	24		203	184
G002	51	45		176
W106	16	28	49	

SNPs are above the diagonal, Indels below the diagonal.

**Table 3:** **Nonsynonymous and synonymous mutations among four *Buchnera *** **Mp strains**

	**USDA**	**F009**	**G002**	**W106**
USDA		59	43	11
F009	69		62	57
G002	63	65		41
W106	18	69	62	

Nonsynonymous substitutions above diagonal, synonymous substitutions below diagonal.

Overall, comparison of the four *Buchnera* Mp genomes shows that the two tobacco-adapted strains (USDA and W106) are more closely related to one another than to F009 and G002 (Tables 3 and 2). This is consistent with the hypothesis that the ability to feed efficiently on tobacco arose relatively recently in *M. persicae* and has spread around the world in the past few decades [30,42]. However, given that the overall gene content of the four *Buchnera* Mp strains is the same and only one gene central to the symbiosis shows a possible functional difference between tobacco and non-tobacco strains of *Buchnera* Mp, it is unlikely that changes within the *Buchnera* genome account for the ability of the aphids to feed from tobacco, thus any advantage *Buchnera* may provide to tobacco adapted lineages would need to result from differential gene expression. That said, previous analyses of global transcription in *Buchnera* demonstrate only a weak or limited capacity for transcriptional regulation of gene expression [43-48] and thus, we suggest that the ability of some *M. persicae* lineages to feed on tobacco results from differences in aphid genome architecture.

**Table 4:** **Comparison of genomic features of four *Buchnera *** **Mp strains**

	** Tobacco**		** Non-tobacco**	
	**USDA**	**W106**	**F009**	**G002**
**Chromosome**				
Genome Size (bp)	643526	643502	643510	643517
Genic Size (bp)	578745	579254	579211	579216
Intergenic Size (bp)	64781	64248	64299	64301
Genic G + C (%)	26.34	26.34	26.34	26.34
Intergenic G + C (%)	15.72	15.68	15.67	15.67
Protein coding genes (#)	581	581	581	581
Pseudogenes (#)	11	11	12	11
tRNA (#)	32	32	32	32
rRNA (#)	3	3	3	3
Avg. gene length (bp)	988.92	988.96	988.89	988.9
Avg. intergenic length (bp)	118.83	118.1	118.2	118.2
Fold genome coverage	248.51	195.94	130.71	375.71
**pLeu**				
Plasmid size (bp)	7802	7802	7800	7799
Protein coding genes (#)	7	7	7	7
**pTrp**				
Fragment size (bp)	2438	2437	2438	2438
Protein coding genes (#)	2	2	2	2

“#” indicates a gene/RNA count for an entity category, which distinguishes from other categories measuring length with unit of bp and G+C content (%).

### *The* Buchnera *Mp genome*, *the largest* Buchnera *genome to date*, *retains metabolic capabilities lost in other* Buchnera *lineages*

**Figure 2:**
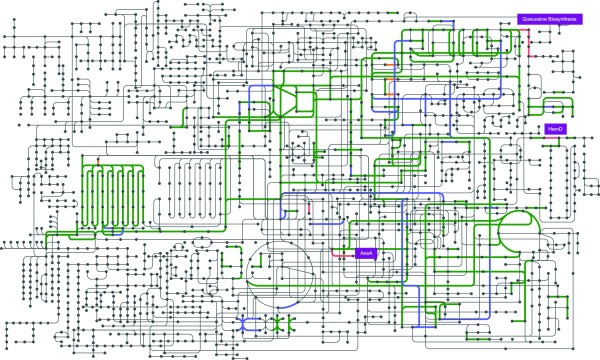
**Map of global KEGG metabolic pathway present in *Buchnera *Mp showing genes present in *Buchnera *Mp but absent in *Buchnera *APS (salmon color), genes with nonsynonymous mutations among the four *Buchnera *Mp strains (blue color), genes with nonsynonymous mutations among the four *Buchnera *Mp strains that are also absent in Buchnera APS (orange color).** Map generated from genome sequence of BuMP_USDA.

The *Buchnera* Mp genome shares features that have previously been described for other *Buchnera* species [16-18,49-51], including a great reduction in genome size and number of genes relative to *Escherichia coli*, low GC content, and a strong bias toward the use of AT-rich codons in coding regions. With 643.5 kb and 579 predicted genes, *Buchnera* Mp is the largest sequenced *Buchnera* genome. *Buchnera* Mp retains more genes homologous to *E. coli* and has more protein coding genes than all other sequenced *Buchnera* genomes. Of particular interest is the fact that the gene profile of *Buchnera* Mp differs from those of other sequenced *Buchnera* strains. Of the 581 coding sequences of *Buchnera* Mp 96% (561) are orthologous to genes found in the second-largest *Buchnera* strain, *Buchnera* APS from *A. pisum*, an aphid that feeds on legumes. The remaining 4% correspond to 21 coding sequences (Additional file 1: Table S2, Figure 2). These 21 genes include two groups that are of particular biological interest: (1) a cluster of queuosine biosynthesis genes, and (2) two protein coding genes that are very rare among all sequenced *Buchnera* genomes. The queuosine biosynthesis cluster comprises five genes (equivalents of *E. coli* genes queC, ygcF(queE), queF, sscR, and folE, Figure 2) required for the biosynthesis of queuosine, a modified tRNA nucleoside found in bacteria and eukaryotes. Although absence of the five queuosine biosynthesis genes in *Buchnera* APS shows that they are not vital for *Buchnera per se*, genes homologs to all five of these genes are found in *Buchnera* Sg from *S. graminum* (AE013218.1), an aphid that feeds on monocots. Animals are unable to synthesize queuosine and must obtain it from their diets. However, given that it is not known whether queuosine can be transferred from *Buchnera* to its host, it is not clear whether queuosine biosynthetic capability impacts the symbiosis of *Buchnera* with their host aphids.

The two protein coding genes that are very rare among all sequenced *Buchnera* genomes are a predicted uroporphyrinogen III synthase (*HemD*, EC 4.2.1.75, USDA_CDS_00013) and a predicted asparaginase (*ansA*, EC 3.5.1.1, USDA_CDS_00060). Uroporphyrinogen III synthase is part of the incomplete heme biosynthetic pathway of *Buchnera*. There is no functional *HemD* encoded in the genome of *Buchnera* APS. However, BLASTp analysis shows the presence of pseudogenized *HemD* in *Buchnera* from *A. pisum* strains LSR1 (NZ_ACFK01000001.1), TLW03 (NC_017252.1) and JF99 (NC_017253.1), suggesting a relatively recent loss of this enzymatic function. The retention of an incomplete heme biosynthesis pathway across all sequenced *Buchnera* genomes shows that the host must supply metabolic precursors for these co-factors. Like other *Buchnera*, the *Buchnera* Mp genome encodes *CysG*, which catalyzes the multistep conversion of uroporphyrinogen III into siroheme, an essential cofactor for sulfite reductase and nitrite reductase. However, due to the presence of *HemD* (uroporphyrinogen III synthase) *Buchnera* Mp can receive metabolic precursors from its aphid host at an earlier step in the pathway. Since the *Buchnera* Mp genome encodes sulfite reductase, but not nitrite reductase, the main function of siroheme synthesis is likely the assimilation of inorganic sulfur, making the presence of siroheme essential for the production of cysteine and methionine [52].

*ansA*, which encodes an asparaginase, is only known from one other *Buchnera* genome, that of *Acyrthosiphon kondoi* (NC_017256.1). As one of the most abundant phloem transport amino acids in plants [53], asparagine should be utilized efficiently in the aphid-*Buchnera* system. Current models for amino acid biosynthesis in *A. pisum* indicate that aphid asparaginase converts asparagine to ammonia and aspartate, which is transported into *Buchnera* APS for the biosynthesis of threonine and lysine [52,54,55]. However, since *Buchnera* does not encode asparagine biosynthesis enzymes, there must be at least some transport of this amino acid from the aphid to the endosymbionts. The presence of *ansA* in the *Buchnera* Mp genome indicates that phloem-derived asparagine may be transported directly to *Buchnera* Mp for the biosynthesis of other amino acids. Similar to the three predicted *A. pisum* asparaginases (XP_001944435, XP_001949831, and XP_001942778), three *M. persicae* ESTs (EE262575, ES22392, and ES223192) arise from genes that encode predicted asparaginases. Therefore, this enzymatic function is not missing from *M. persicae*, despite the fact that it also is encoded by the endosymbiont.

To date, the only significant transcriptional regulation of *Buchnera* amino acid biosynthesis was observed for the methionine biosynthesis pathway of *Buchnera* Sg [45]. This regulatory activity was ascribed to the *metR*, one of the few bacterial transcriptional regulators retained in the *Buchnera* genome. However, although *metR* is present in *Buchnera* Sg and *Buchnera* Mp, it is not found in the other *Buchnera* genomes (Additional file 1: Table S2). *S. graminum* and *M. persicae* have wider host ranges than *A. pisum*, *Cinara cedri*, and *Baizongia pistaceae*. As such, they would be exposed to a broader range of plant sulfur-transport metabolites and might have a greater need to regulate the biosynthesis of methionine. For instance, glutathione and *S*-methylmethionine are the two most abundant forms of reduced sulfur in plant phloem [56]. However, whereas *S*-methylmethionine can likely be converted to methionine by predicted aphid homocysteine methyltransferases, breakdown of glutathione releases cysteine, which would need to be converted to methionine by the *Buchnera* endosymbionts. Therefore, biosynthesis of methionine in *Buchnera* would need to be regulated based on the relative abundance of glutathione and *S*-methylmethionine in different host plants.

### The Buchnera *Mp genome contains a leucine and a tryptophan biosynthesis plasmid*

The plasmid pLeu was assembled and annotated completely for the four *Buchnera* Mp strains, and the gene profile is similar to that of *Buchnera* APS (Table 4). The plasmid pTrp, which in *Buchnera* characteristically contains a repeat of the tryptophan operon [16], was partially assembled in the four *Buchnera* Mp strains. The gene content and gene order of pTrp matches that of *Buchnera* APS. Therefore these two plasmids seem to be fulfilling their roles in the *Buchnera* Mp strains without further variation and evolution.

## Conclusion

Our sequencing of the *Buchnera* endosymbiont genomes from four strains of *Myzus persicae* shows 100% conservation of gene content. This lack of differences between tobacco-adapted and non-adapted strains of *Buchnera* Mp suggests that the endosymbionts play no role in the ability of some *M. persicae* lineages to grow well on tobacco. The presence of *metR* and *ansA* in the genome of *Buchnera* Mp, but not in the genome of *Buchnera* APS, may allow more adaptive use of dietary amino acids and contribute to the ability of *M. persicae* grow on a wider variety of host plants than the legume specialist *A. pisum*.

## Materials and methods

### Selection of aphid lineages

We sequenced the *Buchnera* genome from two isofemale lineages of *M. persicae* collected from tobacco (USDA [from greenhouse tobacco in Ithaca, NY, 2003, 57]) and W106 (from field-grown tobacco in Windsor, CT, 2007)) and two *M. persicae* isofemale lineages from non-tobacco hosts (G002 (from field-grown pepper in Geneva, NY, 2003), and F009 (from field-grown potato in Freeville, NY, 2003; both from [57]). Since their collection, aphid lineages have been maintained in the laboratory on cabbage seedlings (*Brassica oleracea* var. Wisconsin Golden Acre) at 20°C under long day conditions (16 hours light: 8 hours dark). To ensure the maintenance of clonal lineages, microsatellite genotyping is performed biannually (Additional file 1: Table S1).

### Microsatellite genotyping of aphid lineages

Aphid cultures in the lab of Alex Wilson are genotyped biannually to ensure maintenance of clonal integrity. *Myzus persicae* cultures are genotyped at six loci: M40 (forward primer labeled with VIC, M40-for-VIC) and M63 (M63-for-6-FAM) from [58]; myz2 (reverse primer labeled with PET, myz2–rev-PET), myz9 (myz9-for-VIC), myz25 (myz25-for-NED), and S17b (S17b-for-NED) from [59]. All six loci are amplified in a single multiplexed PCR reaction containing 1 × Qiagen PCR Buffer, 2 mM MgCl_2_, 0.21 mM of each dNTP, 1 unit of Qiagen *Taq* DNA polymerase, 0.04 μM of each M40f/r, myz9f/r, myz25f/r, and S17bf/r, 0.08 μM of each myz2f/r, 0.17 μM of each M63f/r and 10 ng of genomic DNA to a final volume of 12 μL. The following touchdown PCR program is run: 94°C for 2 min, followed by 8 cycles of 94°C for 30 sec, touch-down annealing for 1 min at 62°C stepping down 1°C per cycle for 8 cycles, 72°C for 45 sec, followed by 22 cycles of 94°C for 30 sec, 55°C for 1 min, 72°C for 45 sec with a final extension of 72°C for 6 min. PCR products are diluted 1:50 prior to analysis on an Applied Biosystems 3130*xl* Genetic Analyzer using the Applied Biosystems GeneScan 500 LIZ Size Standard.

### *Isolation of* Buchnera *DNA*

*Buchnera* are obligately symbiotic bacteria that have never successfully been cultured. Thus, prior to DNA extraction aphid material was subject to enrichment for intact *Buchnera*. From each lineage, 1.5-2.5 g of aphids was crushed in a cold mortar in 20 ml Buffer A (25 mM KCl, 35 mM Tris–HCl, 100 mM EDTA, 250 mM sucrose, pH 8.0). The homogenate was passed twice through a 100 μm pore-sized nylon filter (Millipore) and centrifuged at 1,500 g at 4°C for 10 min. The pellet was resuspended in 25 ml Buffer A and passed through decreasing pore-sized filters: once through a 20 μm nylon filter (Millipore), twice through an 11 μm nylon filter (Millipore), twice through an 8 μm polycarbonate filter (Whatman, Schleicher & Schuell), three times through a 5 μm polycarbonate Isopore filter (Millipore) and three times through a 3 μm polycarbonate filter (Whatman, Schleicher and Schuell). The filtrate was centrifuged at 1,500 g at 4°C for 25 min and the pellet resuspended in 3 ml Buffer A, and further purified using a Percoll gradient (5% PEG6000, 1% BSA, 1% Ficoll, 250 mM sucrose with 27 or 70% Percoll) by centrifugation at 12,000 g at 4°C for 15 min. *Buchnera* cells were recovered from between the 27 and 70% Percoll layers, diluted in 10 ml Buffer A and centrifuged at 1,500 g at 4°C for 15 min. The cell pellet was washed twice with 2 ml Buffer A. Following washing DNA was extracted from the cell pellet using a Qiagen DNeasy column with inclusion of the RNase A treatment and eluted twice into 100 μl Qiagen Buffer AE yielding an average of 12 μg of *Buchnera* DNA per gram of aphid fresh weight.

To verify our extraction protocol was sufficiently enriching for *Buchnera* DNA relative to aphid DNA, we utilized real time quantitative PCR (RT-qPCR) to measure the abundance of *Buchnera* single copy gene *dnaK* (using primers dnaK2F 5′-GATTGTCTTCGGCTGTTG-3′ and dnaK2R 5′- GTCACTCCTTTATCACTTGG-3′) relative to the abundance of aphid single copy gene *elongation factor 1*α (using primers AWRT002F 5′- CTGATTGTGCTGTGCTTATTG-3′ and AWRT002R 5′- CAAGGTGAAAGCCAATAGAGC-3′). We found that we can reliably increase *Buchnera* copy number in our enriched sample relative to DNA extracted from whole aphids by 100 fold. Meaning that in any given sample we conservatively estimated that *Buchnera* DNA would represent 15% of the reads. An estimate we then used to determine the amount of sequencing that would be required to generate high quality genomes for each of the four *Buchnera* Mp lines.

### *Next*-*generation genome sequencing of* Buchnera aphidicola *Mp*

For each *Buchnera* strain we generated 118 to 124 Mb of DNA sequence data using Roche 454 LR70 sequencing performed by the Environmental Genomics Core Facility at the University of South Carolina, and 136 to 375 Mb of DNA sequence data using an Illumina Genome Analyzer at the Cornell University Life Sciences Core Laboratories Center. DNA for Roche 454 sequencing was prepared for sequencing following the manufacture’s instructions. DNA for Illumina sequencing was randomly sheared by nebulization prior to library preparation using an Illumina Genomic DNA sample preparation kit according to the manufacture’s protocol.

### Verification of genome sequence and assembly by Sanger sequencing

Target fragments in regions with less than 21× Illumina coverage were amplified using the following touchdown PCR program: 94°C for 2 min, followed by 8 cycles of 94°C for 30 sec, touch-down annealing for 1 min at 62°C stepping down 1°C per cycle for 8 cycles, 72°C for 45 sec, followed by 31 cycles of 94°C for 30 sec, 55°C for 1 min, 72°C for 45 sec with a final extension of 72°C for 6 min. PCR amplification was performed in 10 μL reactions containing 1 × Qiagen PCR Buffer, 0.25 mM of each dNTP, 0.5 μM of each primer, 0.2 units of Qiagen *Taq* DNA Polymerase, and 10 ng of genomic DNA. PCR products were purified in 7 μL reactions using 0.1 units of Exonuclease I and 0.1 units of Shrimp Alkaline Phosphatase at 37°C for 15 min followed by enzyme deactivation at 80°C for 15 min. Cycle sequencing using the Applied Biosystems BigDye® Terminator v3.1 Cycle Sequencing Kit was performed in 10 μL reactions with 2 μL of purified PCR product, 0.375 μL BigDye® Terminator v3.1, 1× Sequencing Buffer, and 1 μM of primer under the following thermal cycling conditions: 35 cycles of 94°C for 30 sec, 50°C for 15 sec, and 60°C for 4 min. DNA sequencing reactions were purified using Sephadex G-50 Fine DNA Grade, dehydrated and resuspended in 10 μL Applied Biosystems Hi-Di™ Formamide prior to analysis on the Applied Biosystems 3130*xl* Genetic Analyzer.

### Genome assembly

Roche 454 reads were assembled using Roche Genome Sequencer FLX software. Facilitated by the fact that genome evolution in *Buchnera* is characterized by conservation of gene order [19], we built a genome scaffold for each *Buchnera* Mp strain by BLASTing assembled 454 contigs against the *Buchnera* APS genome (NC_002528). Each scaffold contained gaps. Gap size was inferred by the distance between contigs assembled against the *Buchnera* APS genome. Illumina reads were assembled across gaps using the overlap-consensus approach [60]. Briefly, a 30 bp fragment with a high sequencing quality score from the 3′ end of each contig upstream of a gap was selected as the assembly seed. All Illumina reads and their complementary sequences were maintained in a repository for Illumina read assembly. The repository was searched for Illumina reads containing a 30 bp fragment matching the seed, and up to 2 mismatches were allowed in the matching process. A base after the matched 30 bp fragment of each Illumina read was recorded, and the consensus nucleotide was determined by the majority rule on the basis of recorded bases of all matched reads. The matched reads (and corresponding complementary sequences) from which the last base was recorded were removed from the repository, and not used for further assembly. The assembled consensus sequence was extended by adding the determined consensus nucleotide to 3′ end, and the seed was shifted a base to the 3′ end on the extended sequence after the addition of a base. This process was repeated until the consensus sequence reached the 5′ end of the other contig downstream of the gap.

We used MAQ [61] to map Illumina reads on the assembled sequences with up to 3 mismatches. The coverage of Illumina reads on each site was calculated and used to identify potential sequencing errors. Regions with low coverage of Illumina reads (<21×) were first screened by an in-house PERL script for sequencing errors, such as an indel or a wrong base call originated from Roche 454 reads. Errors attributed to the 454 sequence were corrected using the consensus sequence of Illumina reads. In regions where the errors couldn’t be corrected by the script due to the insufficient Illumina coverage, a Sanger PCR sequencing approach was applied.

### Genome annotation

Gene models in the assembled genomes and plasmid scaffolds were predicted with FgenesB (http://www.softberry.com, utilizing the annotated genome of *Buchnera aphidicola* Bp (NC_004545) as a training set), GeneMark [62], and GLIMMER [63]. Predictions shared by all three gene prediction pipelines were considered candidate genes and annotated by running BLAST against the NCBI non-redundant protein databases (nr) and *Buchnera aphidicola* str. APS (taxid: 107806) proteins. All candidate genes with e-values less than 4e-04 to known proteins were considered *Buchnera* Mp coding sequences and annotated accordingly. Transfer RNAs (tRNAs) were predicted using the online version of tRNAscan [64], whereas rRNAs were annotated by BLAST search.

### Manual curation

All genes that differed in length from the *Buchnera* APS models by >10%, together with any gene models that were interrupted by premature stop codons, were manually curated. These included 38 gene models across the four *Buchnera* Mp genomes. Assessment of automated gene model predictions during manual curation involved alignment of *Buchnera* Mp models against each other, as well as assessment of the similarity, nucleotide and amino acid percent identity and model length against those of *Buchnera* APS and the top *E. coli* hits. In some instances, it was necessary to perform Pfam domain searches [65] to confirm homology. Frequent manual curation issues included: *Buchnera* Mp models that were longer than *Buchnera* APS models, but close in length to the *E. coli* gene; these *Buchnera* Mp models were left unchanged. *Buchnera* Mp models that were longer than both *Buchnera* APS and *E. coli* homologs; these models were also left unchanged. *Buchnera* Mp models that were shorter than the *Buchnera* APS homolog; these were frequently extended to an upstream alternate start codon (often GTG and TTG) and occasionally the rare ATT start codon. Most commonly, manual curation involved indels in polyA tracts resulting in automated annotation either truncating the gene or splitting it into two separate partial gene models. In all cases, DNA sequence on either side of the polyA tract was conserved, and every indication was that the gene had been subject to purifying selection, remaining functional. In these cases, the length of the polyA tract was left unchanged but the gene models were merged or extended, resulting in a DNA sequence that codes for a gene in two reading frames. This feature of symbiotic bacterial genomes, including the genome of *Buchnera aphidicola* from *Schizaphis graminum* has been reported previously [66].

### Pathway analysis

The functional annotation of the verified *Buchnera* Mp genes was performed using the KEGG Annotation Database (http://www.genome.jp/tools/kaas). We compared the query genes against the manually curated KEGG genes database and assigned KO (KEGG Orthology) to query genes. The information of pathways and associated KO were retrieved from KEGG web sites, and the candidate genes were grouped into KEGG pathways accordingly. The number of pathways, as well as the number of genes in each pathway, was counted for each strain. The pathway statistics of the four *Buchnera* Mp strains were compared with the other *Buchnera* strains and *E. coli*, to investigate the evolutionary patterns of pathways in our strains.

### Analysis of non-synonymous mutations

The impact of non-synonymous mutations on proteins was predicted by PROVEAN [39]. The distributions of non-synonymous mutations across the W106, G002 and F009 genomes relative to USDA were tested for randomness [38]. The randomness of nonsynonymous mutations was evaluated by the runs.test function [67] in R [68].

## Competing interests

The authors declare that they have no competing interests.

## Authors’ contributions

ACCW & GJ conceived of and designed the study. ZJ assembled and annotated the genomes and performed bioinformatic analyses in collaboration with SK, NT and ACCW. DHJ performed gap-closing Sanger sequencing and executed genome quality control analysis in collaboration with ACCW. TW isolated and prepared *Buchnera* DNA for genome sequencing in collaboration with ACCW. ACCW & ZJ drafted the manuscript. ACCW, SK, NT and GJ contributed to preparation of the final manuscript. All authors read and approved the final manuscript.

## Authors’ information

ZJ, SK and NT are computational scientists interested in genome assembly using next generation sequencing data and gene prediction. GJ studies insect/host plant interactions with a particular focus on insect elicitors of host defensive responses. DHJ worked as a technician in Wilson’s lab. TW was a graduate student research assistant in Wilson’s lab. Utilizing a diversity of experimental approaches from stable isotopes through whole genome sequencing, ACCW studies the metabolic collaboration of host/symbiont systems with a focus on nitrogen metabolism.

## Supplementary Material

Additional file 1 **Table S1.** Collection information and microsatellite genotype of *Myzus persicae* lineages hosting the four sequenced *Buchnera* Mp strains. **Table S2**. Twenty-one *Buchnera* Mp coding sequences whose counterparts are absent in *Buchnera* APS. **Table S3**. Thirteen nonsynonymous mutations in the *Buchnera* Mp genomes of lineages W106, F009 and G002 that are predicted to be deleterious. **Figure S1**. Nucleotide composition of all protein coding sequences in the genomes of *Buchnera* Mp USDA (BTI), *Buchnera* APS, *Buchnera* Cc and *E. coli*. Data shown by codon position and by all positions. Click here for file
