# Supplementary material for: Comparative analysis of genome sequences from four strains of the Buchnera aphidicola Mp endosymbion of the green peach aphid, Myzus persicae
Source: BMC Genomics. 2013 Dec 24;14:917. doi: 10.1186/1471-2164-14-917 (PMC3890641; doi:10.1186/1471-2164-14-917)
Supplement: Additional file 1 — Table S1. Collection information and microsatellite genotype of Myzus persicae lineages hosting the four sequenced Buchnera Mp strains. Table S2. Twenty-one Buchnera Mp coding sequences whose counterparts are absent in Buchnera APS. Table S3. Thirteen nonsynonymous mutations in the Buchnera Mp genomes of lineages W106, F009 and G002 that are predicted to be deleterious. Figure S1. Nucleotide composition of all protein coding sequences in the genomes of Buchnera Mp USDA (BTI), Buchnera APS, Buchnera Cc and E. coli. Data shown by codon position and by all positions. [file 1471-2164-14-917-S1.docx]

**Supporting Information**

**Table S1. Collection information and microsatellite genotype of *Myzus persicae* lineages hosting the four sequenced *Buchnera* Mp strains.**

|  |  |  |  | Microsatellite fragment size (bp) | | | | | | |
| --- | --- | --- | --- | --- | --- | --- | --- | --- | --- | --- |
| Line | Locality | Date | Host | M40 | M63 | myz2 | myz9 | myz25 | S17b |  |
| USDA | Ithaca, NY | 2003 | tobacco | 112 120 | 171 190 | 193 203 | 214 218 | 118 118 | 166  166 |  |
| W106 | Windsor, CT | 08/2007 | tobacco | 130 130 | 171 177 | 181 193 | 206 208 | 118 118 | 166 166 |  |
| F009 | Freeville, NY | 08/2003 | potato | 124 124 | 177 177 | 189 191 | 210 224 | 116 118 | 166 166 |  |
| G002 | Geneva, NY | 08/2003 | pepper | 120 124 | 192 192 | 179 179 | 204 224 | 116 118 | 162 162 |  |

**Table S2. Twenty-one *Buchnera* Mp coding sequences whose counterparts are absent in *Buchnera* APS**

| CDS | length (aa) | KO title | Kegg Pathway | | orthology in other species* | | | | | |
| --- | --- | --- | --- | --- | --- | --- | --- | --- | --- | --- |
|  |  |  |  |  | **Bp** | **Cc** | **Sg** | **Tuc7** | **5A** | ***E. coli*** |
| USDA_CDS_00013 | 252 | E4.2.1.75, hemD, UROS; uroporphyrinogen-III synthase [EC:4.2.1.75] | Porphyrin and chlorophyll metabolism | | _ | _ | _ | _ | _ | hemD |
| USDA_CDS_00060 | 321 | E3.5.1.1, ansA, ansB; L-asparaginase [EC:3.5.1.1] | Alanine, aspartate and glutamate metabolism; Cyanoamino acid metabolism; Nitrogen metabolism | | _ | _ | _ | _ | _ | ansA |
| USDA_CDS_00121 | 230 | queC; queuosine biosynthesis protein QueC | |  | _ | _ | BUsg463 | _ | _ | queC |
| USDA_CDS_00182 | 128 | E4.2.3.12, ptpS; 6-pyruvoyl tetrahydrobiopterin synthase [EC:4.2.3.12] | Folate biosynthesis | | _ | _ | BUsg402 | _ | _ | sscR |
| USDA_CDS_00183 | 219 | queE, ykvL, ygcF; queuosine biosynthesis protein QueE | |  | _ | _ | BUsg401 | _ | _ | ygcF |
| USDA_CDS_00188 | 196 |  |  | | bbp370 | _ | BUsg396 | _ | _ | ygfA |
| USDA_CDS_00246 | 316 | fabD; [acyl-carrier-protein] S-malonyltransferase [EC:2.3.1.39] | Fatty acid biosynthesis | | bbp320 | BCc_216 | BUsg338 | _ | _ | fabD |
| USDA_CDS_00275 | 322 | znuA; zinc transport system substrate-binding protein | ABC transporters | | _ | _ | BUsg309 | _ | _ | znuA |
| USDA_CDS_00285 | 219 | cmk; cytidylate kinase [EC:2.7.4.14] | Pyrimidine metabolism | | bbp287 | _ | _ | _ | _ | cmk |
| USDA_CDS_00298 | 262 | queF; 7-cyano-7-deazaguanine reductase [EC:1.7.1.13] | |  | _ | _ | BUsg288 | _ | _ | queF |
| USDA_CDS_00300 | 410 | ABC.LPT.P, lolC, lolE; lipoprotein-releasing system permease protein | ABC transporters | | _ | _ | BUsg286 | _ | _ | lolE |
| USDA_CDS_00348 | 156 | E3.1.26.4A, RNASEH1, rnhA; ribonuclease HI [EC:3.1.26.4] | DNA replication | | bbp229 | _ | BUsg239 | _ | _ | rnhA |
| USDA_CDS_00367 | 334 | apbE; thiamine biosynthesis lipoprotein | |  | bbp209 | _ | _ | _ | _ | apbE |
| USDA_CDS_00380 | 305 | E6.3.2.4, ddlA, ddlB, ddl; D-alanine-D-alanine ligase [EC:6.3.2.4] | D-Alanine metabolism | | bbp196 | _ | BUsg208 | _ | _ | ddlB |
| USDA_CDS_00427 | 312 |  |  | | bbp155 | _ | BUsg160 | _ | _ | alx |
| USDA_CDS_00458 | 221 | E3.5.4.16, folE; GTP cyclohydrolase I [EC:3.5.4.16] | Folate biosynthesis | | _ | _ | BUsg129 | _ | _ | folE |
| USDA_CDS_00556 | 301 | metR; LysR family transcriptional regulator, regulator for metE and metH | |  | _ | _ | BUsg030 | _ | _ | metR |
| USDA_CDS_00566 | 337 |  |  | | bbp022 | _ | BUsg020 | _ | _ | yjeK |
| USDA_CDS_00586 | 54 |  |  | | _ | _ | _ | _ | _ | _ |
| USDA_CDS_00590 | 144 |  |  | | _ | _ | _ | _ | _ | _ |
| USDA_CDS_00591 | 42 |  |  | | _ | _ | _ | _ | _ | _ |

* Bp = *Buchnera aphidicola* str. Bp from *Baizongia pistaciae*, Cc = *Buchnera aphidicola* BCc from *Cinara cedri*, Sg = *Buchnera aphidicola* from *Schizaphis graminum*, Tuc7 = *Buchnera aphidicola* str. Tuc7 from *Acyrthosiphon pisum*, 5A = *Buchnera aphidicola* str. 5A from *Acyrthosiphon pisum.*

**Table S3. Thirteen nonsynonymous mutations in the *Buchnera* Mp genomes of lineages W106, F009 and G002 that are predicted to be deleterious.**

| **CDS** | **Mutation** | **PROVEAN Score** | **length** | **name** | **KO title** | **Kegg Pathway** |
| --- | --- | --- | --- | --- | --- | --- |
| W106_CDS_00395 | A637S | -2.876 | 764 | mrcB | mrcB; penicillin-binding protein 1B [EC:2.4.1.129 3.4.-.-] | Peptidoglycan biosynthesis |
| F009_CDS_00045 | S26L | -2.618 | 152 | dut | E3.6.1.23, dut; dUTP pyrophosphatase [EC:3.6.1.23] | Pyrimidine metabolism |
| F009_CDS_00125 | D101E | -2.822 | 208 | clpP | clpP, CLPP; ATP-dependent Clp protease, protease subunit [EC:3.4.21.92] |  |
| F009_CDS_00285 | T197S | -3.331 | 219 |  | cmk; cytidylate kinase [EC:2.7.4.14] | Pyrimidine metabolism |
| §F009_CDS_00295 | S15L | -3.611 | 247 | ybgI |  |  |
| F009_CDS_00355 | M784I | -3.529 | 1161 | dnaE | DPO3A1, dnaE; DNA polymerase III subunit alpha [EC:2.7.7.7] | Purine metabolism; Pyrimidine metabolism; DNA replication; Mismatch repair; Homologous recombination |
| *F009_CDS_00403 | D65N | -2.799 | 428 | thrC | E4.2.3.1, thrC; threonine synthase [EC:4.2.3.1] | Glycine, serine and threonine metabolism; Vitamin B6 metabolism |
| F009_CDS_00419 | R463S | -4.87 | 708 | pta | E2.3.1.8, pta; phosphate acetyltransferase [EC:2.3.1.8] | Taurine and hypotaurine metabolism; Pyruvate metabolism; Propanoate metabolism |
| F009_CDS_00552 | V716A | -3.239 | 1342 | rpoB | rpoB; DNA-directed RNA polymerase subunit beta [EC:2.7.7.6] | Purine metabolism; Pyrimidine metabolism; RNA polymerase |
| G002_CDS_00189 | P176T | -7.679 | 247 | yggJ | rsmE; ribosomal RNA small subunit methyltransferase E [EC:2.1.1.-] |  |
| G002_CDS_00247 | I199T | -2.756 | 316 |  | fabD; [acyl-carrier-protein] S-malonyltransferase [EC:2.3.1.39] | Fatty acid biosynthesis |
| §G002_CDS_00296 | S15L | -3.611 | 247 | ybgI |  |  |
| G002_CDS_00359 | M38T | -3.267 | 251 | uppS | uppS; undecaprenyl diphosphate synthase [EC:2.5.1.31] | Terpenoid backbone biosynthesis; Biosynthesis of terpenoids and steroids |
| *G002_CDS_00404 | D65N | -2.799 | 428 | thrC | E4.2.3.1, thrC; threonine synthase [EC:4.2.3.1] | Glycine, serine and threonine metabolism; Vitamin B6 metabolism |

* A mutation shared by the F009 and G002 *Buchnera* Mp genomes. The other 12 deleterious mutations are found in only one genome but note that § marks mutations in the same gene in the F009 and G002 *Buchnera* Mp genomes.

**Figure S1. Nucleotide composition of all protein coding sequences in the genomes of *Buchnera* Mp USDA (BTI), *Buchnera* APS, *Buchnera* Cc and *E. coli*.** Data shown by codon position and by all positions.
